# Mind-body practices, interoception and pain: a scoping review of behavioral and neural correlates

**DOI:** 10.1080/07853890.2023.2275661

**Published:** 2023-11-08

**Authors:** Stephanie Voss, Daniel A. Boachie, Norberto Nieves, Neha P. Gothe

**Affiliations:** a Department of Kinesiology and Community Health, University of IL Urbana-Champaign, Champaign, IL, USA; b Occupational Therapy, Shirley Ryan Ability Lab, Chicago, IL, USA; c Beckman Institute for Advanced Science and Technology, University of Illinois Urbana Champaign, Champaign, IL, USA; d Bouvé College of Health Sciences, Northeastern University, Boston, MA, USA

**Keywords:** Yoga, mindfulness, mind-body practices, chronic pain, interoception

## Abstract

**Objective:**

Chronic pain is a significant source of suffering in the United States, and many individuals increasingly turn towards yoga for pain relief. However, little is known regarding how yoga improves pain. Herein we seek to examine the scope of the literature linking mind-body practices, pain and interoception; an emerging mechanism by which yoga may improve chronic pain.

**Methods:**

This scoping review followed the five-stage methodological framework proposed by Arksey and O’Malley to examine behavioral and neural correlates of interoception in mind-body practices and pain. A broad search of the Pubmed, CINAHL, SportDiscus, Scopus, PsychInfo, and SocIndex databases was conducted, utilizing three clusters of search terms: (1) interoceptive terms, (2) mind-body terms, and (3) pain terms.

**Results:**

A combined total of 690 articles were screened, and 24 findings included for analysis. Sixteen studies examined interoceptive outcomes in response to mind-body practices for chronic pain, and 8 studies examined interoceptive outcomes in response to evoked-pain tasks in experienced mind-body practitioners. Only three studies linked yoga, interoception and pain.

**Conclusion:**

This review relied on the broader mind-body literature to inform our analyses as the literature examining yoga, pain and interoception remains limited. Interoceptive techniques including attending to and acceptance of bodily sensations, appear to be key therapeutic mechanisms in mind-body practices for chronic pain. Future yoga-based interventions would benefit examining interoceptive outcomes and integrating interoceptive strategies to facilitate the pain-modulating benefits of yoga.

## Introduction

### Background

Chronic pain is a significant source of suffering and disability in the United States, affecting approximately 20% of U.S. adults [1]. As a complex condition with deficits in cognitive appraisal, emotion regulation and physical function, current clinical practice guidelines recommend a multimodal rehabilitation approach to chronic pain [2], where practices requiring patients to be active in the rehabilitation process, such as exercise and mind-body practices, may be particularly beneficial [3]. Yoga is a multimodal approach uniquely positioned to address the physical, cognitive, and emotional components of chronic pain, through the use of physical postures (*asana*), breathing techniques (*pranayama*), and meditation (*dhyana*), three of the most commonly practiced eight limbs of yoga. Indeed, yoga-based practices are increasingly popular for pain management, and previous findings indicate that yoga is beneficial for chronic pain [4–7]. However, the mechanisms by which yoga improves chronic pain remain to be understood.

Interoception is emerging as a mechanism by which yoga may improve pain and psychological health [8] yet little direct evidence exists in support of this hypothesis, or the effects of yoga on interoception in other populations [9]. While definitions vary [10], interoception is generally defined as the sense of internal bodily signals [11,12] but more broadly conceptualized as a multidimensional construct requiring both the perception of internal sensations as well as the cognitive appraisal and emotional evaluation of those bodily signals [11]. Yoga has previously been conceptualized to facilitate the integration of bottom-up interoceptive input with top-down cognitive and emotional regulatory processes (See 8 for an excellent theoretical framework linking philosophical foundations to modern findings), well-suited to addressing the multidimensional cognitive, emotional and physical symptoms common in chronic pain.

A limitation to the yoga and pain literature is the implicit focus on yoga as an exercise modality, often compared to other forms of physical activity or exercise [4,6,7], and the emphasis on *asana* practice as the active ingredient of yoga-based practices. However, in the Yoga Sutras of Patanjali, an ancient text describing the eight limbs of yoga and providing guidelines for living a meaningful and purposeful life [13], only three of the 196 sutras are dedicated to describing *asana* practice (sutras 2.46–2.48), while the remaining describe primarily contemplative and cognitive techniques. Furthermore, B.K.S. Iyengar highlights the role of *asana* as a means to gain awareness of our innermost states by directing attention towards bodily sensations, thoughts, and emotions that arise during *asana* [14], an undeniably interoceptive process. However, even the most detailed yoga protocols rarely specify the cuing that occurs during *asana*, and rather list the postures used in sequence, along with specific breathing and meditation practices. It is therefore possible that cuing in yoga-based interventions could vary widely, such that a greater emphasis might be placed on proprioceptive cueing (e.g. physical alignment) rather than interoceptive cuing (e.g. noticing sensations). We hypothesize that in addition to regular *pranayama* and *dhyana* practices, the benefits of *asana* practice are not limited to the physiological, but also the interoceptive work that occurs while performing physical postures.

### Identifying the research question

As there is limited evidence to support the benefits of yoga on interoception in chronic pain, this scoping review sought to explore the current literature linking mind-body practices to interoceptive processing and pain. The intention was to identify hypothesis-generating patterns within the mind-body literature to inform the selection of interoceptive techniques (e.g. mindful movement, breath awareness, body scans), common in many mind-body practices, to integrate into targeted yoga-based interventions for chronic pain. Consequently, the mind-body practices included in this review were selected due to their origins in or similarity with yoga, including those contemplative traditions conceptualizing a subtle energetic body [11], regardless of their being movement or meditation-based practices.

The choice of interoceptive outcomes included in this review is intentionally broad, to account for the heterogeneity within the literature regarding terminology and assessment [11,12]. In selecting interoceptive outcomes, we follow the four dimensions of interoceptive processing described by Pollatos & Herbert [12], including: interoceptive accuracy (IAc) – the ability to detect bodily signals, such as a heartbeat; interoceptive sensibility (IS) – the subjective report on bodily states, such as muscle tension; interoceptive awareness (IAw) – both the awareness of and confidence in the accuracy of one’s perception of internal states; and emotional evaluation of interoceptive signals (IE) – the interpretation of and attention paid to bodily signals [12].

Additionally, we examine neural correlates of interoceptive processing with the conceptualization that interoception is a multidimensional construct including the sensory perception of internal states along with the cognitive appraisal and emotional evaluation of those bodily signals [11]. As such, we selected neural correlates relevant to this review to be those representing an interoceptive network of regions identified by Critchley et al. [15]. The somatosensory and insular cortices are proposed to play a central role in receiving raw interoceptive signals and generating subjective feeling states [15,16] and ‘second-order’ regions including the anterior cingulate cortex (ACC), ventromedial and dorsolateral prefrontal cortices (vmPFC, dlPFC) [15] assist in the cognitive regulation and emotional evaluation of internal feeling states [12,15]. We select these regions acknowledging the limitation of assuming the accuracy of these regions as key interoceptive substrates, as the field remains relatively nascent. Therefore, we rely on the authors expertise on the neural regions of interest with the purpose of identifying patterns within the literature that link mind-body practices to behavioral and neural correlates of multidimensional interoceptive processing, including the perception, cognitive appraisal, and emotional evaluation of pain.

## Methods

This scoping review was conducted following the 5-stage methodological framework for conducting rigorous, transparent scoping reviews as suggested by Arksey and O’Malley [17] and updated by Levac and colleagues [18]. We outlined the first stage ‘identifying the research question’ in our introduction. Herein, we report on the next four stages and summarize and report the findings in the results section of this review. A detailed protocol can be made available upon request to the corresponding author.

### Identifying relevant studies

This scoping review used a broad search strategy seeking to link three bodies of literature: interoception, mind-body practices and pain. As such, search terms were constructed in three clusters: (1) interoceptive terms, (2) mind-body terms and (3) pain terms (a detailed search string is included in the Supplementary Material with this manuscript). The cluster of interoception terms was intentionally broad to account for the vast heterogeneity of terminology within the literature. Emotion regulation terms and neural regions were included within this cluster to account for the emotional evaluation dimension of interoception [12] and regions hypothesized to reflect the interoceptive neural network [15]. Mind-body terms initially focused on yoga, mindfulness, and meditation, but tai chi and qigong were later included due to the evolving nature of the scoping review. Arksey and O’Malley suggest that search criteria in scoping reviews be amendable to allow for additional criteria to be added once greater familiarity with the field is reached [17].

The primary search was conducted in June 2022 of the following databases: Pubmed, CINAHL, SportDiscus, Scopus, PsychInfo, and SocIndex. We placed limits and filters for English and Human subjects research, with the remaining exclusion criteria applied during screening and review. We identified duplicates with the de-duplication tool in Mendeley desktop. Two additional searches were conducted in July 2023 to: (1) identify new findings published within the past year, and (2) include the search terms tai chi and qigong as mind-body terms. In addition to the database searches, we scanned the reference lists of included articles.

### Study selection

As the reviewer most familiar with the breadth of the topic, the database searches and abstract/title screening were conducted by the primary author. Data extraction was then conducted by two independent reviewers (first and second authors). The final assessment of inclusion/exclusion criteria was determined by consensus between the primary and senior author.

#### Inclusion and exclusion criteria

We included any peer-reviewed, empirical study written in English that examined interoception outcomes in response to either (1) mind-body practices (MBP) for individuals with chronic pain; or (2) pain-evoked tasks in experienced mind-body practitioners. Studies were excluded if they did not meet the inclusion criteria as defined in Table 1. Interoception outcomes were defined as standardized behavioral measures examining changes in self-reported awareness of bodily sensations or changes in at least one of the neural correlates identified by Critchley et al. [15] and listed in Table 1. Mind-body practices were defined as any style of yoga, mindfulness, meditation, tai chi, or qigong.

**Table 1.** Inclusion and exclusion criteria.

General criteria	
Inclusion	Exclusion
English	Non-English
Peer Reviewed Articles Published in a peer reviewed journal	Non-peer reviewed. Dissertations/theses, poster/oral presentations, white papers, book chapters
Original Quantitative Empirical Studies Randomized Controlled, Non-Randomized, Crossover, Longitudinal, Cross-sectional	Non-empirical, Review or Qualitative Literature Review, Narrative Review, Hypothesis and Theory articles, Systematic Reviews, Meta-analyses, Case Studies (*N* < 5)
Interoception Outcomes, at least one: Behavioral: MAIA, BRQ, BAQ, heartbeat tracking or detection tasks. Neural Correlates: somatosensory, somatomotor cortices, insular cortex, anterior cingulate cortex, ventromedial and dorsolateral prefrontal cortices.	No Interoception Outcome as defined. *If included only for a neural correlate, excluded if no pain-related outcome (e.g. working memory in chronic pain).*
Type of Mind-Body Practice Any type of yoga, mindfulness, meditation, *tai chi, or qigong.*	Any other type of Mind-Body Practice e.g. Feldenkrais, Basic Body Awareness Therapy, Psychotherapies without a mindfulness-based component (CBT, ACT), *MBP combined with another intervention (e.g. tDCS).*
Criteria specific to mind-body practices for chronic pain	
Chronic Pain Population Any type of pain lasting ≥ 3 months *(If duration unreported, met by reported diagnostic criteria that includes minimum 3 months pain duration (e.g. fibromyalgia or cLBP))*	Non-Chronic Pain Population Healthy participants, clinical conditions with unreported chronic pain (e.g. Cancer, PTSD), or pain duration < 3 months *(If duration unreported, reported diagnostic criteria that did not include 3 months duration (e.g. some findings in knee osteoarthritis)*
Criteria specific to pain-evoked tasks in mind-body practitioners	
Experienced Practitioners As defined by the original authors.	Non-experienced Practitioners *Healthy practitioners assigned to an MBP intervention.*
Included a pain-evoked task	No pain-evoked task

*Italics* indicate additional criteria added after initial abstract/title screening to limit the scope of the review for more meaningful analysis. MBP: mind-body practice; MAIA: Multidimensional Assessment of Interoceptive Awareness; BAQ: Body Awareness Questionnaire; BRQ: Body Responsiveness Questionnaire; CBT: Cognitive Behavioral Therapy; ACT: Acceptance and Commitment Therapy; tDCS: transcranial direct current stimulation; cLBP: chronic low back pain; PTSD: post-traumatic stress disorder.

Depending on the study population (chronic pain or mind-body practitioners) we defined the following additional criteria. Chronic pain population met by diagnostic criteria (e.g. fibromyalgia, chronic low back pain (cLBP)) or a minimum pain duration of 3 months. Experienced mind-body practitioners are defined by the original authors’ criteria for including individuals with expertise or experience in a mind-body practice deemed sufficient for comparison to a matched novice or naïve control group.

Following the initial abstract/title screening, the primary and senior author revised the inclusion/exclusion criteria (indicated in Table 1) prior to data extraction to include the following additional criteria to narrow the scope of the review for a more meaningful analysis. This revision of criteria was conducted in accordance with Arksey and O’Malley’s suggestion that scoping review criteria be amendable once greater familiarity with the field is reached [17]. Studies included only for neural correlates of interoception were required to include at least one pain outcome as neural correlates of interoception may also activate in response to non-pain related tasks (e.g. working memory). Mind-body practices were further refined to include tai chi and qigong, and excluded any interventions in which a MBP was combined with another intervention (e.g. transcranial direct current stimulation) that could confound the findings. In studies that did not report duration of chronic pain, studies were included if the population’s diagnostic criteria included a minimum duration of at least 3 months of pain (e.g. fibromyalgia, cLBP) but excluded if the pain population included a mix of acute and chronic pain or met diagnostic criteria that did not specify a minimum pain duration of 3 months (e.g. some findings in knee osteoarthritis). We excluded studies examining the relationship between mind-body practices and pain in healthy, practice-naïve individuals assigned to an intervention [19,20] to reflect findings that neural changes in mind-body practitioners may be due to years of regular practice [21,22], and evidence that chronic pain is associated with neuroplastic changes distinct from typical pain processing in healthy individuals [23].

## Charting the data

The following data was extracted from each of the articles reviewed, utilizing Microsoft excel to organize the data in two separate sheets for studies examining interoception outcomes in response to either (1) MBP for individuals with chronic pain; or (2) pain-evoked tasks in experienced mind-body practitioners.

The following data was extracted from all studies: author(s), publication year, sample size, mean age, female to male ratio, type of MBP, control group (if applicable), study design, interoceptive outcomes matching inclusion criteria, remaining outcome measures, and key findings as interpreted by the original authors. Additional data extracted from studies examining individuals with chronic pain included: pain population, minimum pain duration and MBP intervention duration. Additional data extracted from studies in experienced practitioners included: length of experience and type of evoked pain stimulus. Key findings were further separated into behavioral and neural correlates. As the focus of this review was on interoception and pain, we charted significant and nonsignificant results for all interoception and pain-related outcomes. All remaining outcomes examined (e.g. depression, physical function) were charted if significant to provide relevant contextual findings.

## Collating, summarizing and reporting the data

To collate and summarize the findings reported in the results below, the data was organized into two sections: (1) MBP for individuals with chronic pain; or (2) pain-evoked tasks in experienced mind-body practitioners. Within each section, the data was further summarized by (a) behavioral interoception outcomes and (b) neural correlates of interoception. Behavioral outcomes were further subdivided by assessment type and neural correlates were subdivided into task-evoked findings (e.g. neural activity during pain or meditation) and structural/resting state connectivity findings. Based on the patterns that emerged from the literature, we discuss the neural findings within two broad categories: (1) neural processing in regions involved in the perception and awareness of internal bodily signals, and (2) neural processing in regions associated with the cognitive and emotional evaluation of internal states.

## Results

### Articles retrieved

**Figure 1.**
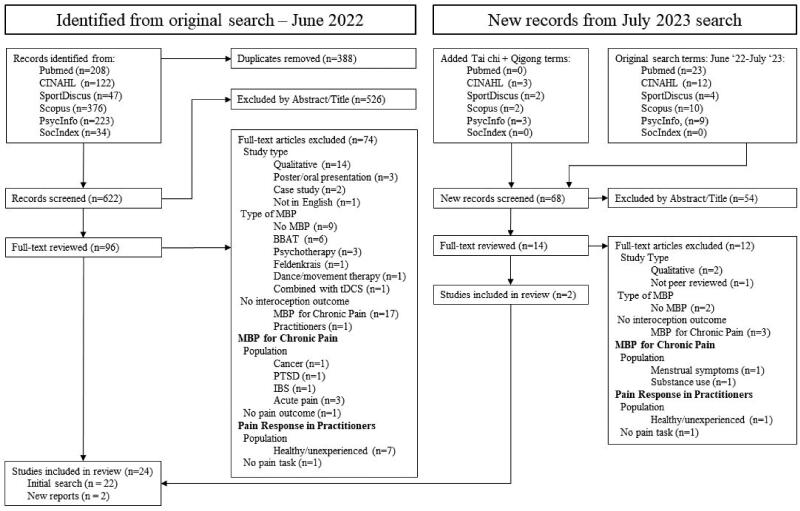
PRISMA flowchart.

A combined total of 690 articles were screened and 24 articles included for analysis. A PRISMA flow diagram of the search procedure is presented in Figure 1. The initial search yielded 1010 hits, with 622 titles remaining after duplicates were removed. All titles and abstracts were examined for relevance and after removing 526 irrelevant hits, 96 titles were reviewed in their entirety and screened for inclusion or exclusion. Seventy-four articles were excluded for reasons listed in the PRISMA flow diagram (Figure 1) and the initial search yielded 22 articles included in this scoping review. The reference lists of these articles were scanned but did not yield any new findings. The additional searches in July 2023 yielded a combined total of 68 new hits, of which 54 were irrelevant. Fourteen were reviewed in their entirety, 12 excluded for the reasons listed in Figure 1, and two additional studies (two tai chi studies from 2016/17) were included for a combined total of 24.

### Effects of MBP on interoception in chronic pain

Sixteen studies examined interoceptive outcomes in response to MBP in chronic pain; study characteristics are detailed in Table 2. Intervention lengths ranged from 2 to 16 weeks and types of chronic pain included: chronic low back (cLBP) [28–30], heterogeneous pain populations [5,31], opioid-treated chronic pain [32,33], fibromyalgia [34,35], chronic neck pain [24,36], knee osteoarthritis [37], chronic musculoskeletal pain [38], migraine [39], and breast cancer survivors with chronic neuropathic pain [40] or persistent post-surgical pain [41]. Types of MBP included: tai chi [24,34,36,37], yoga [5,30], qigong [41], and the following mindfulness-based interventions: brief self-compassion training [28], mindfulness-based stress reduction (MBSR) [29,35,39,40], mindfulness-oriented recovery enhancement (MORE) [32,33], mindfulness-based cognitive therapy (MBCT) [31], and a mindfulness-based pain management program (MBPM) [38]. Key findings for all studies are detailed in Table 3 and summarized below.

**Table 2.** Study characteristics of Mind-Body practices (MBP) for chronic pain.

Author (year)	Sample size; Population; Pain duration; Mean Age; Female: Male	Practice Type; Control group	Study design; Intervention length	Study Outcomes
**Behavioral outcomes**				
de Jong (2016)	*N* = 31; chronic pain + comorbid depression Pain duration: > 3 months Age: 49.5 ± 10.6 23F:8M	MBCT; TAU	Pilot RCT; 8 weeks	**MAIA,** PCS, Quick Inventory of Depressive Symptomatology – Clinician Rated (QIDS-C_16_)
Lauche (2016)	*N* = 114; chronic neck pain Pain duration: > 3 months Age: 49.4 ± 11.7 91F:23M	Tai Chi; Neck Exercises; Wait-list control	RCT; 12 weeks	**MAIA,** VAS-Pain intensity, VAS-Pain on Movement (POM), PSS, HADS, Neck Disability Index (NDI), SF-36 Mental & Physical Health indices, Questionnaire on the Assessment of Physical Wellbeing, Postural Awareness Scale (PAS)
Lauche (2017)	*N* = 75; chronic neck pain Pain duration: > 3 months Age: 49.5 ± 11.6 59F:16M	Tai Chi; Neck Exercises	Secondary analysis of parent RCT [24]; 12 weeks	**MAIA,** VAS-Pain intensity, PSS, HADS, Postural Awareness Scale (PAS)
Osypiuk (2020)	*N* = 21; breast cancer survivors with persistent post-surgical pain Pain duration: >3 months Age: 54 ± 10.2 21 F:0M	Multimodal Qigong Mind-Body Exercise (QMBE); no control	Longitudinal pilot study; 12 weeks	**MAIA,** BPI, HADS, PSS, PCS, Functional Assessment of Chronic Illness Therapy-Fatigue (FACIT-F), Rosenberg Self Esteem Scale (RSE), Godin Leisure Time Exercise Questionnaire (GLTQ), Self-Efficacy for Exercise Scale (SEES), Functional Assessment of Cancer Therapy Breast Symptom Index (FACT-*B* + 4), Grip strength, Shoulder ROM
Roberts (2021)	*N* = 95; opioid-treated chronic pain Pain duration: 201.5 ± 139.4 months Age: 56.8 ± 11.7 63 F:32M	Mindfulness-Oriented Recovery Enhancement (MORE); Support group psychotherapy	Secondary analysis of parent RCT [25]; 8 weeks	**MAIA,** Emotion Regulation Questionnaire (ERQ), Depression Anxiety Stress Scale (DASS)
Schmid (2019)	*N* = 67; heterogenous chronic pain Pain duration: > 6 months Age: 50.78 ± 10.43 46F:21M	Hatha Yoga; Usual care	Pilot RCT; 8 weeks	**BRQ,** BPI, Rand 36-item Health Survey, Chronic Pain Self-Efficacy Scale (CPSS), Stanford Self-Efficacy for Managing Chronic Disease (SSMCD-6)
Sherman (2013)	*N* = 192; cLBP Pain duration: > 3 months Age: 49 ± 9.3 123 F:69M	Viniyoga; Stretching; Self-care book	Secondary analysis of parent RCT [6]; 12 weeks	**8 Questions from BAQ and BRQ combined**, RDQ, PSS, TSK, Arthritis Self-Efficacy Scale, SF-36 Mental health index, Positive States of Mind Scale, Sleep Quality – single item from RDQ, hours of back exercise in past week
**Neural Correlates**				
Berry (2020)	*N* = 20, cLBP Pain duration: 10.66 ± 8.98 years Age: 40.15 ± 12.56 13F:7M	Brief Self-compassion Training; no control	Longitudinal; 8 contact hours over 2 intensive group trainings + 2 weeks home practice	**Pain-evoked fMRI** **MAIA,** PCS, RMQ, PROMIS-Intensity, Self-Compassion Scale (SCS)
Braden (2016)	*N* = 26, cLBP Pain duration: not reported; recruited from spine center where regularly seen by physicians. Age: 44.5 ± 6.9 17F:9M	Abbreviated MBSR; Stress reduction reading	Pilot trial (pseudo-randomized); 4 weeks	**Emotion-evoked fMRI** BDI-II, STAI – State only, Oswetry Low Back Pain Scale
Brown (2013)	*N* = 28; chronic musculoskeletal pain (57% fibromyalgia) Pain duration: not reported; diagnostic criteria from medical records. Age: 46.5 ± 11 21 F:7M	Mindfulness Pain Management Program (MBPM); TAU	RCT; 8 weeks	**Pain-evoked EEG** MPQ-Short form, SF-36 Mental & Physical health indices, Pain Stages of Change Questionnaire, Survey of Pain Attitudes, Mindful Attention and Awareness Scale (MAAS)
Hatchard (2021)	*N* = 21; breast cancer survivors with chronic neuropathic pain Pain duration: > 6 months Age: 52.4 ± 9.7 21F:0M	MBSR; Waitlist control	Sub-study of larger RCT [26]; 8 weeks	**Emotion-evoked fMRI** BPI, FFMQ
Hudak (2021)	*N* = 62; veterans with opioid-treated chronic pain Pain duration: 16.4 ± 12.9 years Age: 59.3 ± 9.9 9F:53M	Mindfulness-Oriented Recovery Enhancement (MORE); Support group psychotherapy	Mechanistic sub-study of larger clinical trial (ongoing); 8 weeks	**EEG during meditation** Opioid dose, Nondual Awareness Dimensional Assessment (NADA-state), Perceived Body Boundaries Scale (PBBS)
Kong (2019)	*N* = 41; fibromyalgia Pain duration: not reported, met fibromyalgia diagnostic criteria. Age: 53 ± 11.3 39 F:2M	Tai-chi; Healthy control	Partial Crossover; 12-weeks	**Resting state fMRI** FIQR, BDI-II
Medina (2022)	*N* = 46; fibromyalgia Pain duration: > 1 year since fibromyalgia diagnosis. Age: 52.6 ± 8.6 46F:0M	MBSR + TAU; FibroQOL + TAU; TAU control	Sub-study of larger RCT [27]; 8 weeks	**ASL, whole brain rCBF maps** VAS-Pain intensity, FIQR, HADS, PCS
Seminowicz (2020)	*N* = 98; migraine Pain duration: ≥ 1 year Median Age: 36 (18–65) 89 F:9M	Enhanced MBSR (MBSR+); Stress Management for Headache	RCT; Weekly for 8 weeks + biweekly for additional 8 weeks (16 weeks total)	**Structural MRI, resting state fMRI, task-evoked fMRI** Headache frequency and intensity (pain diary), Headache Impact Test (HIT), Response to treatment (≥50% reduction in headache days)
Shen (2021)	*N* = 12; postmenopausal women with knee osteoarthritis (KOA) Pain duration: not reported, met by diagnostic criteria Age: 64.5 ± 6.7 12 F:0M	Tai Chi; no control	Exploratory pilot study; 8 weeks	**Structural MRI, resting state fMRI, diffusion tensor imaging (DTI)** VAS-Pain intensity, BPI, Western Ontario and McMaster Universities Osteoarthritis Index (WOMAC)

N: number included in analyses. **Bold text** represents interoception outcomes. Commonly used abbreviations are as follows: ASL: Arterial Spin Labelling; BAQ: Body Awareness Questionnaire; BDI-II: Beck’s Depression Inventory; BPI: Brief Pain Inventory; BRQ: Body Responsiveness Questionnaire; cLBP: chronic low back pain; EEG: Electroencephalography; FFMQ: Five Facet Mindfulness Questionnaire; FIQR: Revised Fibromyalgia Impact Questionnaire; (f)MRI: (functional) magnetic resonance imaging; HADS: Hospital Anxiety and Depression Scale; MAIA: Multidimensional Assessment of Interoceptive Awareness; MBCT: Mindfulness Based Cognitive Therapy; MBSR: Mindfulness Based Stress Reduction; MPQ: McGill Pain Questionnaire; PCS: Pain Catastrophizing Scale; PROMIS: Patient Reported Outcomes Measurement Information System; PSS: Perceived Stress Scale; rCBF: regional Cerebral Blood Flow; RCT: Randomized Controlled Trial; RMQ/RDQ: Roland-Morris Low Back Pain and Disability Scale; ROM: Range of Motion; SF-36: Short Form 36 Item; STAI: State Trait Anxiety Inventory; TAU: Treatment as Usual; TSK: Tampa Scale for Kinesiophobia; VAS: Visual Analog Scale.

**Table 3.** Effects of Mind-Body Practices (MBP) on interoceptive outcomes in chronic pain.

Author (year)	Key findings
**Behavioral Outcomes**	
de Jong (2016)	Mindfulness-Based Cognitive Therapy (MBCT) vs. Treatment as Usual (TAU) in chronic pain with comorbid depression. Pre-training: **MAIA-ND did not correlate with other MAIA subscales.** *Distraction from pain coping style may be distinct from other aspects of body awareness in chronic pain*. Post-training: **MBCT vs. TAU: ↑MAIA-SR; ↑MAIA-ND partially mediated ↓depression; No effect on MAIA-N, MAIA-AR** *MBCT improved self-regulatory capacity and may enhance ability to turn towards, rather than away from pain. Learning coping styles in contrast to distraction/avoidance may be an important predictor of mental health outcomes in chronic pain.* **MBCT within group: ↑MAIA-EA** *MBCT improved awareness of connection between body and emotional states.*
Lauche (2016)	Tai Chi vs. Neck Exercises vs. Waitlist control in chronic neck pain. Post-training: **Tai Chi vs. Waitlist: ↑MAIA (mean),** ↓VAS-Pain intensity, ↓Neck Disability (NDI), ↑SF-36, ↓Pain on Movement *Tai Chi improved pain intensity, evoked pain on movement, neck-related disability, and quality of life. Interoceptive sensibility demonstrated a mean increase after training.* **Tai Chi vs. Neck Exercises: No differences between groups.** *Tai Chi had a comparable effect to neck exercises on interoceptive sensibility and all other outcomes.*
Lauche (2017)	Secondary analysis of parent RCT [24]; Examined predictors of reduced pain intensity in Tai Chi vs. Neck Exercises. Regression analysis **Tai Chi & Neck Exercises**: **MAIA (ns),** ↑Baseline VAS-Pain intensity, ↓HADS-A, ↑postural awareness predicted 40.6% of variance in reduced pain. *Regardless of intervention group, higher baseline pain, anxiety reduction and improved postural awareness, but not interoceptive sensibility, predicted reductions in pain after treatment.*
Osypiuk (2020)	Longitudinal Qigong Mind-Body Exercise in breast cancer survivors with persistent post-surgical pain. Post-training: **Qigong: ↑MAIA-NW, ↑MAIA-AR, ↑MAIA-EA, ↑MAIA-SR, ↑MAIA-BL, ↑MAIA-TR, MAIA-N (ns), MAIA-ND (ns),** ↓BPI-Severity, ↓BPI-Interference, ↑FACIT-F, ↑FACT-B, ↓HADS, ↓PSS, ↓PCS, ↑RSE *Qigong improved several clinical outcomes, including pain severity/interference, fatigue, quality of life, mood, stress, pain catastrophizing and self-esteem as well as several domains of interoceptive sensibility except noticing and not distracting.*
Sherman (2013)	Secondary analysis of parent RCT [25]; Examined MAIA-SR as a mediator of emotion regulation after Mindfulness-Oriented Recovery Enhancement (MORE) vs. Support Group (SG) Psychotherapy in opioid-treated chronic pain. Post-training: **MORE vs. SG: ↑MAIA-total, ↑MAIA-SR,** ↑ERQ, ↓DASS. *MORE had a greater effect on improving overall interoceptive sensibility, interoceptive self-regulation, cognitive reappraisal, and emotional distress than psychotherapy.* Mediation analysis: **MORE vs. SG: ↑MAIA-SR mediated ↑ERQ**; ↑ERQ mediated ↓DASS. *MORE facilitated reductions in emotional distress by improving cognitive reappraisal via enhanced interoceptive self-regulation.*
Schmid (2019)	Hatha Yoga vs. Usual Care in heterogenous chronic pain Post-training: **Yoga vs. Usual care: ↑BRQ,** ↑CPSS (total, physical function, & pain management) *Yoga had a greater impact on improving body responsiveness and self-efficacy for pain management than usual care.* **Yoga within group: ↑BRQ,** ↓BPI-Interference, ↑CPSS, ↑SSMCD-6; Both groups: ↑Rand-36 QOL *Both groups improved overall quality of life, only yoga also improved pain interference in addition to improved body responsiveness and pain self-efficacy.*
Sherman (2013)	Secondary analysis of parent RCT [6]; Examined mediators of improved back function in Yoga vs. Stretching in cLBP. Mediation analysis: **Yoga**: 61% ↓RDQ explained by: **↑**self-efficacy, hours of exercise, **↑BRQ/BAQ**, ↑SF-36-MCS, ↓PSS, ↓RDQ-Sleep item **Stretching:** 50% ↓RDQ explained by: **↑**self-efficacy, hours of exercise, **↑BRQ/BAQ**, ↑SF-36-MCS, ↓TSK, ↑Positive states of mind *Self-efficacy and hours of exercise most significantly mediated improved back function in both groups. Body awareness was not an independent mediator for either group. Qualitative data suggested to reflect distinct mechanisms, including greater relaxation/awareness in yoga and greater discipline/routine in stretching.*
**Neural correlates**	
Berry (2020)	Longitudinal Brief Self-Compassion training in cLBP; Pain-evoked fMRI in a self-compassionate state. Post-training, neural correlates: **During pain:** ↓R TPJ ∼↓PROMIS-Pain intensity *Suggested to reflect enhanced present-moment awareness during pain, correlating with reduced pain intensity.* **During pain anticipation: ↑vPCC ∼↑MAIA-BL; ↑R dlPFC** ∼↓SCS (Self-Compassion Scale) *Interpreted as less prefrontal recruitment, reflecting more efficient use of self-compassion resources, and increased awareness of body ownership and internal sensations.* Post-training, behavioral outcomes: **During pain:** VAS-Pain intensity (ns), VAS-Pain unpleasantness (ns) **Self-Compassion training: ↑MAIA-total, ↑MAIA-AR, ↑MAIA-SR, ↑MAIA-BL, remaining MAIA subscales all (ns);** ↑SCS ∼ ↑daily practice; ↓PROMIS-Intensity, ↓RMQ. *Increased self-compassion after training associated with amount of daily practice. Self-compassion training improved pain intensity, disability, and several domains of interoceptive sensibility, despite no difference in evoked pain ratings.*
Braden (2016)	Abbreviated MBSR vs. Stress Reduction Reading (RCon) in cLBP; Emotion-evoked fMRI Post-training, neural correlates: **During sadness-induction task, MBSR vs. RCon: ↑sgACC∼↑sad valence ratings; ↑L vlPFC, ↑dmPFC (trend), ↑R vlPFC (trend)** *Interpreted to reflect increased conscious access to emotional state, corresponding with emotional intensity ratings, and greater prefrontal activity interpreted as increased attentional awareness of emotional state.* Post-training, behavioral outcomes: **During sadness-induction task:** VAS-arousal (ns), VAS-valence (ns) **MBSR:** ↓BDI-II (somatic-affective), ↓ODI; **Stress-Reduction:** ↓BDI (cognitive); **Both groups:** ↓BDI, State Trait Anxiety (STAI) (ns) *Abbreviated MBSR uniquely improved back pain symptoms and somatic-affective component of depression, whereas both groups improved in overall depression with no change in anxiety or evoked emotional arousal or valence ratings.*
Brown and Jones (2013)	Mindfulness-Based Pain Management (MBPM) vs. Treatment as Usual (TAU) in chronic musculoskeletal pain; Pain-evoked EEG Post-training, neural correlates: **During pain, MBPM vs. TAU: ↓aINS (ns),** ↓amygdala (ns) *Interpreted as mildly reduced emotional processing during pain.* **During pain anticipation, MBPM vs. TAU: ↓dlPFC & ↓S2 deactivation (i.e. ↑activity) ∼ ↑Mental health & ↑Pain controllability** *Interpreted as greater cognitive control and focus on body sensations, rather than distraction during pain anticipation, correlated with improved mental health and perceived control of pain.* Post-training, behavioral outcomes: **During pain:** VAS-Pain unpleasantness (ns) **MBPM vs. TAU:** ↑Engagement & ↑Pain controllability predicted ↑SF12-MCS; ↑MAAS, MPQ (ns) *No change in evoked pain ratings or the sensory/affective components of pain. MBPM improved mental health and mindfulness. Suggested that perceived pain control may have a greater impact on mental health outcomes than the sensory pain experience.*
Hatchard (2021)	MBSR vs. Waitlist in breast cancer survivors with chronic neuropathic pain; Emotion-evoked fMRI Post-training, neural correlates: **During emotional Stroop task, MBSR vs. Waitlist: ↓S1, ↓dlPFC,** ↓precuneus, *Interpreted as MBSR reducing activity in regions associated with affective and pain-related words becoming less salient, requiring less executive control over emotions, and less personalized, respectively.* Post-training, behavioral outcomes: **During emotional Stroop task:** Controls ↑reaction time to affective words; MBSR reaction time (ns) *Interpreted as increased emotional reactivity over time without intervention.* **MBSR vs. Waitlist:** ↓BPI-Interference, ↑FFMQ, BPI-intensity (ns) *MBSR improved pain interference and mindfulness despite no change in pain intensity.*
Hudak (2021)	MORE vs. Support Group (SG) Psychotherapy in veterans with opioid-treated chronic pain; Mindfulness-evoked EEG Post-training, neural correlates: **MORE vs. SG: ↑frontal theta power mediated ↓opioid dosing, ↑frontal alpha power, ↑Frontal-Midline Theta (FMT) coherence.** *Interpreted to reflect increased prefrontal activation after MORE, and mindfulness-induced endogenous theta stimulation to PFC improved self-regulation of pain symptoms (i.e. reduce need for opioids). Increased FMT coherence interpreted to reflect enhanced PFC-ACC connectivity, such that mindfulness may facilitate plasticity between top-down and bottom-up self-regulation structures.* Post-training, behavioral outcomes: **MORE vs. SG:** ↓opioid dose, ↑self-referential processing *Mindfulness facilitated reduction in opioid craving and changes in self-referential processing associated with ego-dissolution and sense of oneness between self and world. Suggested that mindfulness shifted valuation from drug-related rewards to internal rewards.*
Kong (2019)	Tai Chi vs. No Control in Fibromyalgia; Fibromyalgia vs. Healthy control at baseline; resting state fMRI Baseline: **Fibromyalgia vs. healthy controls: ↑dlPFC-rACC/mPFC connectivity** *Fibromyalgia demonstrated altered connectivity, suggested to reflect self-regulatory mechanisms to cope with repeated pain.* Post-training, neural correlates: **Tai Chi: ↑dlPFC to rACC/mPFC connectivity ∼ ↓FIQR** *Tai chi further enhanced the altered connectivity in fibromyalgia, associated with clinical improvements, suggested to reflect allostasis such that tai chi further promotes self-regulation to pain.* **Tai Chi: ↓rACC/mPFC connectivity to periaqueductal gray & hippocampus & ↑rACC/mPFC connectivity with postcentral gyrus/operculum,** mid/inferior prefrontal gyrus, and MCC *Tai Chi reduced connectivity of rACC/mPFC to regions associated with pain as a form of long-term learning* [23] *and enhanced connectivity to regions associated with the sensory, cognitive, and emotional aspects of pain.* Post-training, behavioral outcomes: **Tai Chi:** ↓FIQR, ↓BDI-II *Tai chi improved fibromyalgia and depression symptoms.*
Medina (2022)	MBSR vs. Psychoeducation (FibroQOL) vs. TAU in Fibromyalgia; Arterial Spin Labelling fMRI Baseline: **All groups: ↑VAS-Pain intensity predicted ↑ACC and ↑aINS rCBF;** ↑amygdala rCBF ∼ ↑HADS-A, ↑PCS, not VAS-Pain intensity *At baseline, regions associated with emotional processing of pain correlated with greater pain symptoms. Increased amygdala rCBF correlated with negative emotional symptoms but not pain.* Post-training, neural correlates: **MBSR & FibroQOL:** rCBF to ACC & aINS no longer correlated with VAS-Pain intensity; **FibroQOL:** ↓amygdala, no longer correlated with HADS-A and PCS. *Reduction in blood perfusion after training to emotional processing regions suggested to reflect disruption in pain-related processing, and reorganization of salience and pain networks.* Post-training, behavioral outcomes: **MBSR:** ↓PCS – magnification (did not survive correction); **FibroQOL:** ↓PCS – rumination, helplessness; **Controls**: ↓VAS-Pain intensity; **All groups:** HADS (ns), FIQR (ns). *FibroQOL may have a greater impact on appraisal processes related to pain catastrophizing. No effect on symptoms of anxiety, depression, or functional impact in either group. Controls reduced pain intensity, potentially reflecting regression to the mean.*
Seminowicz (2020)	Enhanced MBSR (MBSR+) vs. Stress Management for Headache (SMH) in migraine; Structural MRI, resting state & cognitive task fMRI Post-training, neural correlates: **Both groups:** ↓aMCC GMV, **↓aINS connectivity to cognitive task network** **MBSR + vs. SMH: ↓aINS connectivity to PPC & cuneus; During cognitive task**: ↓cuneus & **↓parietal operculum (including pINS)** *Decreased aINS connectivity to key regions of the cognitive task network interpreted as increased cognitive efficiency after MBSR+, or less depletion of cognitive and emotional resources during pain. Decreased pINS activity during cognitive task interpreted as increased cognitive efficiency to direct cognitive resources to task demands, rather than pain regulation.* Post-training, behavioral outcomes: **MBSR + vs. SMH:** ↓headache frequency (pain diary), ↑response to treatment (52% vs 23% in control), ↓HIT, headache intensity (ns) *MBSR + reduced headache frequency, headache-related disability and had a greater response to treatment, despite no change in headache intensity ratings.*
Shen (2021)	Tai Chi vs. No control in postmenopausal women with knee osteoarthritis; Structural MRI, resting state fMRI, DTI. Post-training, neural correlates: **Tai Chi: ↑mPFC (ns) to amygdala connectivity ∼ ↑physical function; ↑mPFC (ns) to amygdala DTI connectivity ∼ ↓stiffness** *Decreased mPFC-amygdala connectivity previously linked to increased chronic pain symptoms. Moderate, but non-significant increased connectivity after Tai Chi suggested to reflect reversal of chronic pain associated neural changes, associated with improved symptoms.* Post-training, behavioral outcomes: **Tai Chi:** ↓knee pain (VAS, WOMAC and BPI), ↓WOMAC-stiffness, ↑WOMAC-physical function *Tai chi improved pain and stiffness symptoms and improved physical function.*

**Bold text** represents interoceptive outcomes examined in this review. *Italicized text* represents author interpretations of key findings. Abbreviations: 1) Methodological abbreviations: (ns): non-significant; (∼): correlates with; cLBP: chronic low back pain; DTI: diffusor tensor imaging; EEG: electroencephalography; (f)MRI: (functional) Magnetic Resonance Imaging; GMV: gray matter volume; rCBF: regional cerebral blood flow; RCT: Randomized Controlled Trial. 2) Neural regions: aINS/pINS: anterior/posterior insula; aMCC: anterior midcingulate cortex; dlPFC/vlPFC/dmPFC: dorsolateral/ventrolateral/dorsomedial prefrontal cortices; PPC: posterior parietal cortex; S1/S2: primary/secondary somatosensory cortices; sgACC/rACC: subgenual/rostral anterior cingulate cortices; TPJ: temporo-parietal junction; vPCC: ventral posterior cingulate cortex; FMT: frontal-midline-theta coherence. 3) Questionnaires: BAQ/BRQ: Body Awareness/Responsiveness Questionnaires; BDI: Beck’s Depression Inventory; CPSS: Chronic Pain Self-Efficacy Scale; FACIT-F: Functional Assessment of Chronic Illness Therapy-Fatigue; FACT-B: Functional Assessment of Cancer Therapy Breast Symptom Index; FFMQ: Five Facet Mindfulness Questionnaire; FIQR: Revised Fibromyalgia Impact Questionnaire; HADS: Hospital Anxiety and Depression Scale; HIT: Headache Impact Test; MAAS: Mindful Attention and Awareness Scale; MAIA: Multidimensional Assessment of Interoceptive Awareness; MAIA-N: Noticing; MAIA-ND: Not Distracting; MAIA-NW: Not Worrying; MAIA-AR: Attention Regulation; MAIA-EA: Emotional Awareness; MAIA-SR: Self-Regulation; MAIA-BL: Body Listening; MAIA-TR: Trusting; MPQ: McGill Pain Questionnaire; PCS: Pain Catastrophizing Scale; PSS: Perceived Stress Scale; Rand-36: Rand-36 Measure of Health Related Quality of Life; RMQ/RDQ: Roland-Morris Low Back Pain and Disability Scale; RSE: Rosenberg Self Esteem Scale; SF-36 (MCS): Short Form 26 (Mental Component Score); SSMCD-6 = Stanford Self-Efficacy for Managing Chronic Disease; TSK: Tampa Scale for Kinesiophobia; VAS: Visual Analog Scale; WOMAC: Western Ontario and McMaster Universities Osteoarthritis Index.

#### Behavioral outcomes

Eight studies reported behavioral interoception outcomes, of which six used the Multidimensional Assessment of Interoceptive Awareness (MAIA) [24,28,31,33,36,41], one used the Body Responsiveness Questionnaire (BRQ) [5] and one used a subset of questions combined from the BRQ and Body Awareness Questionnaire (BAQ) to examine body awareness [30]. No studies used a heartbeat detection/tracking task to assess interoceptive accuracy.

##### Multidimensional assessment of interoceptive awareness (MAIA)

Four studies identified improvements in interoceptive sensibility assessed with the MAIA in response to MBP in chronic pain, including MBCT [31], qigong [41], MORE [33], and brief self-compassion training [28]. Two reports (from the same trial) found a mean increases in MAIA scores in response to tai chi [24], and reported that interoceptive sensibility was not a predictor of reduced pain [36].

Differences in which MAIA subscales responded to MBP in chronic pain were also reported. Not-distracting and Self-regulation scales improved in response to MBCT, where Not-distracting partially mediated reductions in depression, and changes in Noticing and Attention Regulation were not significant [31]. Total MAIA and Self-regulation improved in response to MORE, and Self-regulation was a significant mediator of cognitive reappraisal, which facilitated reductions in emotional distress [33]. Qigong improved all MAIA domains except Noticing and Not-distracting [41]. Self-compassion training improved total MAIA along with Self-regulation, Body Listening and Attention Regulation, with a trend towards improving Emotional Awareness, Trusting and Not Worrying [28].

##### Body awareness and body responsiveness questionnaires (BAQ, BRQ)

The only two yoga interventions included in this analysis used the BAQ and BRQ [5,30]. Body responsiveness improved in response to Hatha yoga [5] whereas body awareness was not a significant independent mediator of improved back function in response to Viniyoga [30].

#### Neural correlates

Nine studies examined changes in neural correlates of interoception in response to MBP for chronic pain [28,29,32,34,35,37–40]. Behavioral findings and original author interpretations of neural correlates are detailed in Table 3. For brevity, only neural correlates and pain outcomes are summarized below.

##### Task-evoked changes: fMRI and EEG

Six studies examined task-evoked neural activity, of which four used functional magnetic resonance imaging (fMRI) [28,29,39,40] and two used electroencephalography (EEG) [32,38], to examine pain-, emotion-, and attention-evoked activity. The emotion-evoked studies were included as they reported emotion-regulation findings consistent with second-order neural representations of interoceptive processing [15] and the emotional evaluation component of interoception [12].

##### Pain-evoked neural activity

Two studies examined pain-evoked neural activity in response to self-compassion training [28] and MBPM [38]. In both studies, there were no post-training differences in pain-evoked visual analog scale (VAS) intensity or unpleasantness ratings despite improvements in functional pain outcomes. During pain stimulation, self-compassion training produced reductions in right temporo-parietal junction (rTPJ) activity, correlating with reductions in pain intensity [28], and MBPM produced mild, but non-significant, reductions in amygdala and anterior insula (aINS) activity [38]. During pain anticipation, self-compassion training produced increased right dlPFC and ventral posterior cingulate cortex (vPCC) activity correlating with lower self-compassion and greater Body Listening, respectively [28]; and MBPM produced greater dlPFC and somatosensory (S2) deactivation (i.e. increased activity) correlating with improved mental health and pain controllability [38].

##### Emotion-evoked neural activity

Two studies examined emotion-evoked neural activity in response to abbreviated MBSR [29] and MBSR [40]. Both studies found improvements in functional pain outcomes but no change in pain intensity [40] or the sensory/affective aspects of pain [29]. On a sadness-induction task, abbreviated MBSR produced increased subgenual anterior cingulate cortex (sgACC) activity, correlating with emotional valence/intensity ratings, and increased vlPFC and dmPFC activity [29]. On an emotional Stroop task with pain-related sensory and affective words, MBSR produced reductions in S1, precuneus and dlPFC activity [40].

##### Attention-evoked neural activity

Two studies examined attention-evoked neural activity in response to MORE during mindfulness [32] and enhanced MBSR (MBSR+) during a cognitive task [39]. Pain outcomes included reduced opioid usage [32] and reduced headache-related disability with no change in intensity [39]. During meditation, MORE increased frontal alpha and theta power where increased theta power mediated reductions in opioid dosing. MORE also increased frontal-midline-theta (FMT) coherence, reflecting increased prefrontal to limbic structure connectivity [32]. During a cognitive task, MBSR + reduced activity in the cuneus and parietal operculum, a region including the posterior insula [39].

##### Structural and connectivity differences

Four studies examined differences in brain volume or structural and/or functional connectivity. Three examined resting state functional connectivity [34,37,39], one used structural MRI [39], and one used Arterial Spin Labelling [35].

##### Structural MRI

One study examined structural brain changes as part of a multimodal neuroimaging study in response to MBSR + in which there were no significant differences between groups, but both MBSR + and controls demonstrated reductions in anterior midcingulate cortex (aMCC) volume [39].

##### Resting state functional connectivity (rsFC)

Three studies examined changes in rsFC in response to tai chi [34,37] and MBSR+ [39]. In addition to the clinical improvements after MBSR + reported above [39], tai chi improved fibromyalgia [34], and knee pain [37] symptoms. One study noted altered rsFC prior to intervention, where individuals with fibromyalgia had greater dlPFC to rostral anterior cingulate cortex/medial prefrontal cortex (rACC/mPFC) connectivity compared to healthy controls [34]. Tai chi further enhanced this altered connectivity, correlating with fibromyalgia symptom improvements, and reduced rACC/mPFC connectivity to the periaqueductal gray and hippocampus and increased rACC/mPFC connectivity to regions linked to the cognitive, emotional and sensory aspects of pain [34]. In knee osteoarthritis, tai chi produced a non-significant increase in mPFC-amygdala connectivity, correlating with improved physical function [37]. MBSR + demonstrated greater reductions in aINS connectivity to specific regions of the cognitive task network, including the posterior parietal cortex and cuneus [39].

##### Brain perfusion

One study used Arterial Spin Labelling, an fMRI method to generate regional cerebral blood flow (rCBF) maps of subtle blood perfusion differences in response to MBSR [35]. Prior to intervention, greater rCBF to ACC and aINS were associated with greater pain intensity ratings, and greater rCBF to the amygdala correlated with greater anxiety and pain catastrophizing. After training, MBSR did not demonstrate any unique differences in pain outcomes or neural activity, but rCBF to emotional processing regions was no longer associated with pain intensity after MBSR or active the active control intervention (psychoeducation) [35].

### Interoceptive processing of pain in experienced mind-body practitioners

Eight studies examined interoceptive differences in response to evoked-pain in experienced mind-body practitioners; study characteristics are detailed in Table 4. Types of practitioners included: practitioners of the Nyingma and Kagyu traditions of Tibetan Buddhism, similar to mindfulness [42,43], Zen meditation [44,45], Vipassana meditation, a form of mindfulness [46], Transcendental meditation [47], any type of formal meditation practice [48], and any type of yoga practice integrating postures, meditation and breathing [49]. Key findings are detailed in Table 5 and summarized below.

**Table 4.** Study characteristics of interoception outcomes in experienced mind-body practitioners.

Author (year)	Sample size; Experience; Age; Female: Male	Practice Type; Control group	Study design; Pain Stimulus	Study Outcomes
**Behavioral outcomes**				
Zorn (2021)	*N* = 70 (Practitioners *N* = 27) Experience: > 10,000 h Age: 52.6 ± 7.7 34F:36M	Kagyu or Nyingma schools of Tibetan Buddhism; Novice controls with 2-day training and ∼20 h OM/FA.	Cross-sectional; thermal heat	**MAIA,** VAS-Pain intensity, VAS-Pain unpleasantness, PCS, FFMQ, BDI, STAI, Drexel Defusion Scale (DDS), Cognitive Fusion Questionnaire (CFQ), Penn State Worry Questionnaire (PSWQ)
**Neural correlates**				
Brown and Jones (2010)	*N* = 27 (Practitioners *N* = 12) Experience: 39-1820 weeks Age: 34 ± 14 13 F:14M	Any form of formal meditation practice; Naïve controls	Cross-sectional; laser stimuli	**Pain-evoked EEG** VAS-Pain unpleasantness
Gard (2012)	*N* = 34 (Practitioners *N* = 17) Experience: 10.2 ± 6.6 years Age: 37.1 ± 7.8 8F:26M	Vipassana meditation; Naïve controls	Cross-sectional; transcutaneous electrical stimuli	**Pain-evoked fMRI** VAS-Pain intensity, VAS-Pain unpleasantness, VAS-Anticipatory anxiety
Grant (2010)	*N* = 39 (Practitioners *N* = 19) Experience: 14.4 ± 8.4 years Age: 37.6 ± 10.7 9F:30M	Zen meditation; Naïve controls	Cross-sectional; thermal heat	**Structural MRI** Pain sensitivity (temperature to report moderate pain)
Grant (2011)	*N* = 26 (Practitioners *N* = 13) Experience: >1000 h Age: 38.2 8 F:18M	Zen meditation; Naïve controls	Cross-sectional; thermal heat	**Pain-evoked fMRI** VAS-Pain intensity, VAS-Pain unpleasantness
Lutz (2013)	*N* = 28 (Practitioners *N* = 14) Experience: > 10,000 h Age: 45.4 ± 10.7 10F:18M	Kagyu or Nyingma schools of Tibetan Buddhism; Novice controls with 7 days x 30-minute OP/FA home practice.	Cross-sectional; thermal heat	**Pain-evoked fMRI** VAS-Pain intensity, VAS-Pain unpleasantness
Orme-Johnson (2006)	*N* = 24 (Practitioners *N* = 12) Experience: 31.3 ± 2.3 years Age: 57 ± 4.6 12 F:12M	Transcendental Meditation; Novice controls with 4-day training and 5 months home practice.	Longitudinal partial crossover; thermal heat	**Pain-evoked fMRI** VAS-Pain intensity
Villemure (2014)	*N* = 28 (Practitioners *N* = 14) Experience: 9.6 ± 2.8 years Age: 36.8 ± 6.9 18F:10M	All types of yoga integrating physical postures, meditation, and breathing exercises; Naïve controls	Cross-sectional; thermal pain threshold, cold pain tolerance	**Structural MRI** Pain threshold (temperature), Cold pain tolerance (time), VAS-Confidence that yoga improves pain tolerance, Self-reported mental strategies.

N: number included in analyses. **Bold text** represents interoceptive outcomes included in analyses. Commonly used abbreviations are as follows: BDI: Beck’s Depression Inventory; EEG: Electroencephalography; FA: Focused Attention; FFMQ: Five Facet Mindfulness Questionnaire; (f)MRI: (functional) Magnetic Resonance Imaging; OM: Open Monitoring; OP: Open Presence; MAIA: Multidimensional Assessment of Interoceptive Awareness; PCS: Pain Catastrophizing Scale; STAI: State Trait Anxiety Inventory; VAS: Visual Analog Scale.

**Table 5.** Pain-related interoception outcomes in experienced mind-body practitioners.

Author (year)	Key findings
**Behavioral outcomes**	
Zorn (2021)	Buddhist Practitioners vs. Novice Controls; Evoked Pain during an Open Presence Meditative State Behavioral outcomes: **Practitioners vs. Controls during pain:** ↓VAS-Pain unpleasantness, VAS-Pain intensity (ns) **Practitioners vs. Controls: ↑MAIA**, ↑DDS, ↑FFMQ, ↓PCS, ↓CFQ, ↓PSWQ, ↓STAI, ↓BDI **Both groups:** ↑DDS & ↓PCS predicted VAS-Pain unpleasantness, not VAS-Pain intensity. **↓PCS ∼ ↑MAIA,** ↑DDS, and ↑FFMQ *Interoceptive sensibility was not the focus of this analysis, but it was higher in practitioners and negatively correlated with pain catastrophizing across groups. Only cognitive defusion had a higher correlation, and most strongly predicted reduced unpleasantness, suggested to be a core construct in mindfulness-based pain modulation.*
**Neural correlates**	
Brown (2010)	Meditation Practitioners (any type) vs. Naïve Controls; Pain-evoked EEG Neural correlates, Practitioners vs. Controls: **During pain: ↓S2, ↓INS** (did not survive correction for anticipatory activity). **During pain anticipation: ↑mPFC/pACC predicted ↓VAS-Pain unpleasantness, mediated by ↓MCC, inverse pattern to controls where ↑mPFC/pACC predicted ↑VAS-Pain unpleasantness.** Lifetime experience predicted ↓MCC. *Interpreted to reflect reduced negative appraisal of pain, such that increased cognitive control in anticipation of pain reflects greater acceptance of pain, rather than anticipation of threat, contributing to inverse unpleasantness ratings relative to controls.* Behavioral outcomes, Practitioners vs. Controls: Pain threshold: (ns) **During pain:** ↓VAS-Pain unpleasantness (only with 6+ years of experience) ∼ greater experience. *Despite no difference in pain threshold, more experienced practitioners demonstrated lower pain unpleasantness ratings.*
Gard (2012)	Vipassana (mindfulness) Practitioners vs. Naïve controls; Pain-evoked fMRI while in a mindful state Neural correlates, Practitioners vs. Controls: **During pain: ↓bilateral lPFC, ↑pINS/S2 ∼** ↓VAS-Pain unpleasantness **During pain anticipation: ↑rACC/vmPFC** *Interpreted to reflect increased sensory and reduced cognitive processing during pain, and greater attention regulation while anticipating pain. Less unpleasantness correlated with greater sensory pINS/S2 activity in practitioners, but lower activity in controls.* Behavioral outcomes, Practitioners vs. Controls: **During pain:** ↓VAS-Pain unpleasantness (22%), VAS-Pain intensity (ns) **During pain anticipation:** ↓VAS-anticipatory anxiety (29%) *Despite no difference in pain intensity, practitioners reported less unpleasantness and anticipatory anxiety.*
Grant (2010)	Zen Practitioners vs. Naïve Controls; Structural MRI and pain-evoked task Neural correlates, Practitioners vs. Controls: **Cortical thickness: ↑dACC, ↑S2; ↑Years experience ∼ ↑ACC thickness; ↑Hours experience ∼ ↑S1 thickness** Both groups: ↓Pain sensitivity ∼ **↑**ACC, **↑**S2 and **↑**INS thickness *Lower pain sensitivity was associated with greater cortical thickness in affective and pain-related regions, with no differences between groups, despite practitioners demonstrating lower pain sensitivity. Authors suggest a more general relationship between cortical thickness and pain, where greater cortical thickness in practitioners may be the result of long-term practice.* Behavioral outcomes, Practitioners vs. Controls: **Pain Sensitivity**: ↓Pain sensitivity *Practitioners demonstrated a higher pain threshold than controls.*
Grant (2011)	Zen Practitioners vs. Naïve controls; Pain-evoked fMRI, outside a meditative state Neural correlates, Practitioners vs. Controls: **During pain:** ↑thalamus, **↑ACC and ↑INS activation;** ↑hippocampus, ↑amygdala, **↑dlPFC and ↑mPFC deactivation** **Practitioners: ↓dACC to dlPFC connectivity predicted ↓Pain sensitivity.** *Interpreted to suggest that practitioners demonstrate a reduction in appraisal/emotion-related regions and greater activation in primary pain processing regions during pain, and less connectivity between cognitive and pain regions. This ‘functional decoupling’ is suggested to allow painful stimuli to be experienced with more acceptance/cognitive disengagement and less threat.* Behavioral outcomes, Practitioners vs. Controls: **During pain:** VAS-Pain intensity (ns), VAS-Pain unpleasantness (ns) **Pain Sensitivity:** ↓Pain sensitivity *Despite no differences in pain intensity and unpleasantness ratings, practitioners had a higher pain threshold.*
Lutz (2013)	Buddhist Practitioners vs. Novice Controls; Pain-evoked fMRI while in an Open Presence meditative state Neural correlates, Practitioners vs. Controls: **During pain: ↑dorsal aINS/aMCC (salience network), pINS/S2 (ns)** **Pain anticipation: ↓aINS/aMCC,** ↓amygdala *Reduced aINS/aMCC activity during pain anticipation and increased aINS/aMCC activity during pain – components of the salience network assigning emotional weight to homeostatically-relevant/interoceptive stimuli* [50] *– were interpreted to reflect a downregulation of negative anticipatory processing, such that pain is experienced with more acceptance and less unpleasantness.* Behavioral outcomes, Practitioners vs. Controls: **During pain:** ↓VAS-Pain unpleasantness, VAS-Pain intensity (ns) *Practitioners reported lower pain unpleasantness despite similar intensity ratings.*
Orme-Johnson (2006)	Transcendental Meditation Practitioners vs. Novice controls; Pain-evoked fMRI outside a meditative state Neural correlates, Practitioners vs. Controls: **During pain, baseline:** ↓whole brain, ↓thalamus activity **During pain, after 5 months training for novices:** Pain-related activity (ns) between practitioners and controls; Controls ↓thalamus, ↓PFC, ↓whole brain & ↓ACC (ns) activity. *Practitioners only demonstrated reduced pain-related activity prior to novices practicing for 5 months, after which there were no longer and significant differences.* Behavioral outcomes, Practitioners vs. Controls: **During pain:** VAS-Pain intensity (ns) *There were no differences in pain intensity ratings between practitioners and controls at either time point.*
Villemure (2014)	Yoga Practitioners (any type) vs. Naïve controls; Structural MRI and pain tolerance task Neural correlates, Practitioners vs. Controls: **GMV: ↑cingulate/SMA/S1, ↑INS/S2, ↑dmPFC; ↑INS GMV ∼ ↑pain tolerance; ↑left INS GMV ∼ ↑years of experience** **Cortical thickness: ↑cingulate/S1, ↑INS/S2** *Yoga practitioners demonstrated increased GMV and cortical thickness in multiple regions, but only insular GMV correlated with greater pain tolerance. As a primary interoceptive region, authors suggest that greater insular GMV may be the result of regular application of interoceptive strategies from long-term yoga practice..* Behavioral outcomes, Practitioners vs. Controls: **Pain tolerance task:** ↑pain tolerance (2x longer) **Pain threshold:** (ns) **Self-reported mental strategies during pain: Interoceptive/acceptance vs. distraction/avoidance used by controls.** *Despite similar pain thresholds, practitioners demonstrated greater pain tolerance and reported using interoceptive strategies (e.g. acceptance of sensations, focus on the breath) during pain.*

**Bold text** represents interoceptive outcomes examined in this review. *Italicized text* represents author interpretations of key findings. Abbreviations: 1) Methodological abbreviations: (ns): non-significant; (∼): correlates with; (f)MRI: (functional) Magnetic Resonance Imaging; EEG: electroencephalography; GMV: gray matter volume; 2) Neural regions:: aINS/pINS: anterior/posterior insula; dlPFC/vlPFC/dmPFC: dorsolateral/ventrolateral/dorsomedial prefrontal cortices; S1/S2: primary/secondary somatosensory cortices; SMA: supplementary motor area; dACC/rACC/pACC: dorsal/rostral/posterior anterior cingulate cortices, aMCC: anterior midcingulate cortex; 3) Questionnaires: BDI: Beck’s Depression Inventory; CFQ: Cognitive Fusion Questionnaire; DDS: Drexel Defusion Scale; FFMQ: Five Facet Mindfulness Questionnaire; MAIA: Multidimensional Assessment of Interoceptive Awareness; PCS: Pain Catastrophizing Questionnaire; PSWQ: Penn State Worry Questionnaire; STAI: State Trait Anxiety Inventory; VAS: Visual Analog Scale.

#### Behavioral outcomes

Only one study in practitioners use a behavioral interoception measure in which Buddhist practitioners reported lower unpleasantness ratings than controls, despite similar intensity ratings on a pain-evoked task [43]. While interoceptive sensibility (MAIA) was not the focus of this analysis, it was higher in practitioners and negatively correlated with pain catastrophizing across both groups. The primary findings of this study reported that cognitive defusion (the psychological distancing from thoughts and feelings), was the most specific predictor of pain unpleasantness, surviving an adjustment for pain catastrophizing, also strongly associated with unpleasantness ratings [43].

#### Neural correlates

Seven studies examined neural correlates in response to pain-evoked tasks in practitioners to matched novice (no experience) or practice-naïve (minimal training) controls. Behavioral findings along with the original author interpretations of neural correlates are detailed in Table 5. For brevity, only neural correlates and pain outcomes are summarized below.

##### Pain-evoked activity: fMRI and EEG

Five studies examined pain-evoked neural activity: four used fMRI [42,45–47], and one used EEG [48].

##### Pain-evoked activity in a mindful/meditative state

Two studies examined pain-evoked neural activity while in a mindful or meditative state in mindfulness practitioners of the Vipassana [46] and Buddhist [42] traditions. In both studies, practitioners demonstrated lower unpleasantness but similar intensity ratings to controls. During pain, Vipassana practitioners demonstrated greater pINS/S2 activation associated with lower unpleasantness ratings [46] whereas Buddhists demonstrated greater aINS and aMCC activity but no differences in pINS/S2 activation [42]. During pain anticipation, Vipassana practitioners demonstrated lower lPFC and greater rACC/vmPFC activity [46] and Buddhists demonstrated lower aINS, aMCC and amygdala activity [42].

##### Pain-evoked activity outside a mindful/meditative state

Two studies instructed practitioners to not meditate/engage in mindfulness during pain in Zen [45] and transcendental meditation (TM) [47] practitioners. Zen practitioners demonstrated lower pain sensitivity (i.e. required higher stimulus intensity) to match similar subjective pain intensities as controls [45] and TM practitioners demonstrated no difference in pain intensity ratings [47]. Zen practitioners demonstrated greater activation in sensory pain regions including the insula, thalamus and dACC, despite controlling for greater stimulus intensity in practitioners, whereas controls demonstrated greater activation in prefrontal and emotion-processing regions in the bilateral dlPFC, amygdala, left middle frontal gyrus, right hippocampus and mPFC/orbitofrontal cortex [45]. Furthermore, greater meditation experience was associated with greater de-activation in prefrontal regions, and meditators with the lowest pain sensitivity had the weakest functional connectivity between the dACC and dlPFC [45]. TM practitioners demonstrated lower pain-related response in the thalamus and whole brain but no other regions of interest examined, including the ACC and PFC [47]. After novices learned and practiced TM for 5 months, there were no longer any significant differences between groups.

One study instructed participants to focus only on the unpleasantness of pain in meditation practitioners of any discipline [48]. Differences in pain unpleasantness ratings only differed when the sample was reduced to practitioners with a minimum of 6 years of experience, after which lower unpleasantness correlated with greater experience. During pain, practitioners demonstrated lower INS/S2 activity, but this effect did not survive correction for anticipatory activity prior to stimulation. During pain anticipation, practitioners demonstrated lower MCC activity, an effect predicted by greater meditation experience and associated with decreased unpleasantness. Controls demonstrated greater mPFC/pACC activity during pain anticipation, correlating with greater unpleasantness ratings, an effect that was reversed in practitioners and mediated by lower anticipatory MCC activity [48].

##### Structural differences

Two studies used MRI to compare structural differences to performance on pain tasks in Zen [44] and yoga [49] practitioners. Zen practitioners demonstrated greater dACC and S2 cortical thickness, and years of experience correlated with greater ACC thickness. Across participants, lower pain sensitivity was associated with greater cortical thickness in the right dACC, S2 and insular cortices with no differences between groups, despite practitioners demonstrating lower pain sensitivity overall [44]. Yoga practitioners demonstrated greater pain tolerance and greater gray matter volume in multiple cortical regions, including the S2/insular cortices, cingulate/SMA/S1 and left dorsal mPFC [49]. However, only insula volume correlated with increased pain tolerance in practitioners and left insular volume correlated with years of experience [49].

## Discussion

The purpose of this scoping review was to examine the scope of the literature on mind-body practices and pain with a focus on interoceptive outcomes, to inform yoga-based interventions for chronic pain. Patterns of findings are discussed below, with the intention of forming hypotheses for future research on yoga, interoception and pain. Prior to a further discussion of the findings, we note that of the 24 studies included in this analysis, only two were large randomized controlled trials including a behavioral or neural correlate of interoception [24,39]. Interoception was not the primary outcome in either of these trials, and therefore they may not have been sufficiently powered to reliability detect change. Of these two trials, changes in interoception were reported on an exploratory basis in one [24], and analyses of secondary outcomes were not adjusted for multiple comparisons in the other [39], wherein multiple comparisons of secondary outcomes can increase the probability of a false significant result. Furthermore, the remaining intervention trials were secondary analyses, subsets of larger trials or pilot studies designed to evaluate preliminary feasibility, but for which efficacy analyses would require a full randomized controlled trial. Lastly, all the studies on experienced mind-body practitioners were cross-sectional and therefore must also be interpreted with caution. Cross-sectional designs cannot evaluate causation, and are limited to potential bias or selection effects (e.g. individuals with greater pain tolerance or interoceptive awareness may be more likely to choose a MBP) that may influence results. As such, we suggest that these findings must all be interpreted with caution but viewed as informative for future research on yoga, interoception and pain.

### Yoga, interoception and pain

As hypothesized, the current literature linking the three areas of interest for this review – yoga, interoception and pain – is sparse, with only two yoga-based interventions [5,30] and one study of yoga practitioners [49] identified. These findings must also be interpreted with caution as they are either cross-sectional [49], preliminary pilot findings results [5], and/or use body awareness measures [5,30], previously noted to be inadequate for evaluating interoceptive sensibility [51], but included as they provide useful insight into the relationship between yoga, body awareness, and chronic pain.

Findings from the two yoga interventions conflict, where body responsivity improved in one account [5] but was not a significant mediator of improved back function in the other [30]. These could reflect differences in pain population, where Schmid et al. [5] reported a complex, heterogeneous pain population with high opioid use and activity limitations, whereas Sherman et al. [30] only included individuals with cLBP receiving no concurrent pain care and without complex causes of pain. Or, they could reflect the use of the full standardized questionnaire in one finding [5] whereas Sherman et al. [30] acknowledged the limitations of these measures [51] and used a subset of questions from the BRQ and BAQ combined, selecting items most likely to respond to yoga [30]. However, improved interoceptive sensibility in response to yoga in other populations, including healthy sedentary individuals [52], adults with neurodisability [53], and PTSD [54], suggests that it would be an important outcome to examine in future trials of yoga for chronic pain.

The only study to report interoceptive differences in yoga practitioners [49] presents intriguing results as the insular cortex, a primary region in interoceptive processing [15,16] was the only cortical region to mediate improved pain tolerance in practitioners. Additionally, despite no instructions on specific strategy use, practitioners reported using interoceptive strategies during pain, including focused breathing, non-reactive observing of sensations, and acceptance of pain in contrast to distraction and avoidance techniques by controls. Authors suggest that regular yoga practice may promote a more adaptive interoceptive response to pain, contributing to structural differences in the insula [49].

### MBP and self-reported interoceptive sensibility

Patterns of the effects of MBP on interoceptive sensibility in chronic pain began to emerge in the six interventional studies that used the MAIA [24,28,31,33,36,41]. All three of the mindfulness-based interventions improved interoceptive sensibility, of which two demonstrated improvements in comparison to active treatment controls [31,33], and one after longitudinal intervention [28]. In contrast, of the three studies using movement-based MBPs, only qigong improved interoceptive sensibility [41] whereas two reports (from the same trial) did not report significant differences after tai chi [24,36], of which one analyzed the MAIA only on an exploratory basis [24]. However, tai chi did yield a mean increase on all of the MAIA subscales [24] and postural awareness was a significant predictor of reduced pain after tai chi [36]. Within postural awareness, it was specifically the effortless awareness and connectedness score that predicted improved pain, suggested to reflect a more effortless awareness between one’s posture, wellbeing and habitual movement patterns to facilitate pain relief [36]. The discrepancy in these findings could reflect differences in MBP delivery. As described by the authors, the mindfulness-based and qigong interventions all included an emphasis on cognitive appraisal skills [28,31,33,41] in addition to breath and body awareness, whereas the tai chi interventions described a greater emphasis on breath, body and movement awareness but not cognitive skills [24,36]. In accordance with the conceptualization of interoception as a multidimensional construct, we suggest that interoceptive strategies that facilitate not only awareness of, but also appraisal and evaluation of internal signals are essential components of MBP for chronic pain.

Subscale analyses reveal further insight into the benefits of distinct interoceptive techniques. While all of the MAIA subscales were not included in all reports, only Self-regulation [28,31,33,41] and Body Listening [28,33,41] consistently showed improvements in the studies in which they were included. Interoceptive Self-regulation specifically measures the ability to regulate distress by attending to bodily sensations [55] suggesting that training in the ability to use breathing techniques and body awareness to self-regulate distress may be a particularly important therapeutic mechanism of MBP for chronic pain [33]. Improved Body Listening after self-compassion training was also the only scale to correlate with changes in neurological processing during pain anticipation, particularly in the vPCC [28]. Authors interpret this finding to suggest the self-compassion produced a greater awareness of and ability to focus on bodily sensations while anticipating pain, lending support to the above findings that breath and body awareness may be key self-regulatory mechanisms for chronic pain. Finally, one study noted that improvements in Not-distracting mediated reductions in depression symptoms, and was the only subscale to not correlate with any other MAIA scales [31]. These findings could reflect the study’s population (chronic pain with comorbid depression) or, authors suggest that Not-distracting, defined as the tendency to not ignore or distract oneself from sensations of pain or discomfort [55], may represent a coping style distinct from other components of interoceptive sensibility in chronic pain [31]. As we discuss in more detail in regard to neural correlates below, attending to rather than ignoring or distracting oneself from unpleasant sensations appears to be a key technique for regulating the pain experience, as previously noted in mechanistic findings [56,57].

### Neural correlates of interoceptive processing

Herein we briefly summarize the findings on neural correlates of interoceptive processing, relying on the author’s expertise for their interpretation of neural activity. We broadly categorize these findings in two sections: (1) neural processing in regions primarily involved in the perception and awareness of internal bodily signals, including the somatosensory and insular cortices [15,16], and (2) neural processing in regions associated with the cognitive and emotional evaluation of internal signals [12,15], including the anterior cingulate (ACC), ventromedial and dorsolateral prefrontal cortices (vmPFC and dlPFC).

#### Detecting internal states

Experienced practitioners showed some, albeit inconsistent, differences in primary interoceptive processing regions. During pain, increased pINS/S2 activity was associated with reduced pain unpleasantness while in a mindful state [46], but another report found no change in pINS/S2 activity while in engaged in Open Presence (OP) meditation [42]. Rather, OP produced greater aINS/aMCC activity increased during pain and decreased aINS/aMCC activity during pain anticipation [42]. Authors indicate that the aINS/aMCC are key regions of the salience network [42], important for assigning emotional weight to homeostatically-relevant stimuli [50]. As the aINS is proposed to be essential for the subjective awareness of internal sensations [15] whereas the pINS is more involved in perceiving pain intensity [23], these subtle differences could reflect different meditation styles. In mindfulness, attention is directed towards the present moment, including towards the pain experience, along with an attitude of nonjudgmental acceptance [46]. In contrast, OP meditation places greater emphasis on cultivating a state of effortless openness and acceptance [42]. While overlapping, the attention to and acceptance of bodily sensations are distinct interoceptive strategies, both of which may be essential in modulating the pain experience and important to examine into yoga-based interventions for chronic pain.

A few findings noted structural differences in the insula and somatosensory cortices among experienced practitioners, linked to experience and reduced pain sensitivity [44,49], but there were no unique structural differences in response to MBP for chronic pain. Rather, neural correlates in response to MBP in chronic pain occurred primarily in prefrontal and emotion processing regions. These could reflect differences in pain processing in individuals with chronic pain in contrast to healthy individuals [23] or the vast differences in experience, where experienced practitioners had multiple years of regular practice in contrast to 2–3-month intervention periods (Tables 3 & 5). It has been previously suggested that it may take longer to develop more automatic, bottom-up processing of sensory and interoceptive input, but that the integration of bottom-up interoceptive processing with top-down cognitive and emotion-regulation processing may be an essential benefit of yoga-based practices [8]. A few findings begin to lend support to this hypothesis. MBSR + reduced aINS connectivity to regions of the cognitive task network, suggested to reflect improved cognitive efficiency, and less depletion of cognitive and emotional resources during pain [39]. Similarly, MORE increased frontal-midline-theta coherence, interpreted to reflect increased PFC to ACC connectivity, or greater plasticity between top-down and bottom-up self-regulation structures [32].

#### Cognitive and emotional evaluation of pain

As noted above, neural correlates in response to MBP in chronic pain occurred primarily in prefrontal and emotion processing regions, with similarities to correlates in long-term practitioners. MBPM reduced dlPFC and S2 deactivation during pain anticipation, correlating with pain controllability, suggested to reflect a greater ability to maintain cognitive control and focus on bodily sensations [38]. MBSR increased sgACC and prefrontal activity during a sadness-induction task, interpreted as greater access to and attentional awareness of one’s emotional state [29]. MORE produced greater frontal alpha and theta power, interpreted to reflect greater prefrontal activation and improved self-regulation, corresponding with improved self-referential processing [32]. Furthermore, tai chi further enhanced altered connectivity between the dlPFC and rACC/mPFC, associated with clinical improvements, suggested to reflect greater self-regulatory processes to cope with repeated pain [34]. Additionally, tai chi reduced rACC/mPFC connectivity to the periaqueductal gray and hippocampus, important regions in the conceptualization of chronic pain as a form of long term learning [23].

Similar interpretations appear in the findings of experienced practitioners. During pain anticipation, practitioners demonstrated greater mPFC/pACC [48] and rACC/vmPFC [46] activity, predicting reduced unpleasantness in one finding [48], and interpreted to reflect greater attention regulation prior to pain. Similarly, reduced dACC to dlPFC connectivity predicted reduced pain sensitivity, suggested to reflect a functional decoupling to allow painful stimuli to be interpreted with more acceptance/cognitive disengagement than threat [45]. As a whole, the use of cognitive and emotion regulatory interoceptive strategies such as maintaining attention to and acceptance of unpleasant sensations, appear common in mind-body practitioners and may present an important initial therapeutic target for MBP in chronic pain.

### MBP and the pain experience

One of the most interesting patterns that emerged is that MBPs largely have a greater impact on functional pain outcomes, reflecting the ability to effectively manage pain symptoms, despite limited or no improvements in pain intensity. In MBP studies that included both pain intensity and functional pain outcomes, individuals with chronic pain demonstrated no difference in evoked pain intensity and unpleasantness ratings despite improvements on functional pain outcomes [28,38], reductions in pain interference despite no change in intensity ratings [5,40], and reduced migraine frequency and functional impact despite no change in intensity [39]. Additionally, seven of the eight studies examining evoked pain in experienced practitioners reported lower unpleasantness despite similar intensity ratings [42,43,46,48], or higher pain thresholds in practitioners matched to similar subjective intensities [44,45,49]. While the exact neural regions involved continue to be explored, authors do largely concur that regular mind-body practitioners are able to decouple the sensory and cognitive/affective pain experience [45,46], such that greater pain intensity does not always imply a greater negative emotional experience of pain. We suggest that this pattern matches the conceptualization of interoception as a multidimensional construct including sensory perception of internal states along with the cognitive appraisal and emotional evaluation of those bodily signals [11]. MBPs appear to improve the relationship between oneself and unpleasant bodily sensations, such that they are experienced with acceptance rather than avoidance. The ability to maintain focused attention on the breath, sensations and non-judgmental acceptance of the sensory experience emerged as common techniques amongst MBP contributing to an improved pain experience.

### Limitations

This review presents several limitations. First and foremost is the nature of the scoping review, such that the purpose is to examine the scope of the current literature on a nascent body of research, rather than to critically evaluate the findings or draw conclusions [17]. While our primary purpose was to examine the link between yoga, interoception and pain, the yoga-based literature is sparse, and we relied on findings from the wider mind-body literature to inform our review. Within the mind-body literature included, the findings disproportionately examined mindfulness and meditation-based practices. While the MBSR curriculum is rooted in yogic and Buddhist practices [58] and includes yoga postures, many of the other practices included in this review are primarily seated cognitive-based practices (e.g. MBCT, MORE, Zen, TM), and may not confer similar effects as movement-based practices such as yoga, tai chi and qigong. Additionally, most of the findings included in this review must be interpreted with caution due to either small sample sizes or study design, including primarily longitudinal, cross-sectional, and pilot studies, as well as secondary analyses/subsamples of larger RCTs (Tables 2 and 4). Only two large RCTs were identified for this review [24,39], where self-reported interoceptive sensibility was a secondary outcome in only one [24]. Furthermore, we did not a priori specify length of experience in mind-body practitioners required to be included in this review. As such, we included studies with practitioners who’s experience ranged from as little as 39 weeks [48] or 1000 h [45] to some with over 30 years of experience [47].

Another limitation is the interoceptive outcomes chosen for this review. Only seven studies utilized the MAIA [24,28,31,33,36,41,43], currently the most commonly used measure of self-reported interoceptive sensibility [10] and two utilized either the BAQ or BRQ, with previously noted limitations [51]. Additionally, we identified no findings using interoceptive accuracy or a heartbeat detection task as an interoceptive outcome matching our remaining criteria. The effects of MBP on interoceptive accuracy in chronic pain remain to be further examined, with conflicting findings regarding whether interoceptive accuracy is [59] or is not [60,61] altered in individuals with chronic pain. We a priori selected the above measures as interoceptive outcomes for this review, but acknowledge that other measures were identified, including a sensation manikin to assess the ratio of pleasant to unpleasant sensations [62] and a dynamic interoceptive signature scale [63]. Additionally, we selected only those mind-body practices with similar contemplative traditions and overlapping practices to yoga. However, other mind-body practices such as Feldenkrais and Basic Body Awareness Therapy and psychotherapeutic techniques like Cognitive Behavioral Therapy,with potentially distinct or overlapping mechanisms, did appear in our search and would be worth examining in future studies.

Perhaps the most significant limitation is the assumption of an interoceptive neural network. While these regions have previously been linked to interoceptive processing [15] and aligns with interoception as a multidimensional construct linking sensory, affective and cognitive processing of internal sensations [11], this body of literature is still new and much remains to be determined. Additionally, pain appears to be processed differently in individuals with chronic pain [23], such that pain-evoked neural activity in experienced mind-body practitioners may not accurately inform neural processing in response to an MBP intervention in chronic pain. This limitation may further be influenced by the demographic differences between studies in mind-body practitioners in comparison to MBP for chronic pain. Most notably, interventions in chronic pain included predominantly middle-aged to older adult (40–60 years old) female participants, whereas studies in experienced practitioners are predominantly in young to middle aged (30–40 years old) male participants. While women are disproportionately more likely to experience chronic pain and be less physically active [64], they are also more likely to practice yoga [65], suggesting that yoga is a particularly useful modality for chronic pain management. However, in examining the current literature, we acknowledge that pain processing in primarily male practitioners may not accurately inform the female experience of chronic pain in response to MBP.

### Future directions

In its current state, the mind-body literature on interoception and pain exists primarily in seated mindfulness and meditation-based practices. Of the 16 trials examining MBP for chronic pain, only two utilized yoga [5,30], one qigong [41] and two tai chi [24,36] and only one study examined neural correlates of interoception in response to pain in yoga practitioners [49]. Both seated and movement-based MBPs appear to target interoceptive processes and have overlapping techniques, particularly regarding breath awareness, breath regulation and noticing internal sensations, but we hypothesize that movement-based practices such as yoga have the potential to train individuals to adaptively apply interoceptive strategies to a more diverse range of sensory experiences that may be highly relevant to individuals with chronic pain navigating the challenges of their daily routine. The findings by Villemure et al. [49] lend support to this hypothesis, in which experienced yoga practitioners were not instructed on any particular strategy to use during a cold pain tolerance task, yet they still reported the application of interoceptive strategies during pain without being instructed to do so [49]. By challenging practitioners to notice sensations, regulate breathing and adaptively respond to their internal experience from one posture to the next, each yoga posture may provide a new and unique interoceptive experience.

We believe the current state of the literature is too limited to draw conclusions regarding the conferred benefits of seated or movement-based practices on interoception in chronic pain. In our summary of MBP on self-reported interoceptive sensibility above, mindfulness-based practices [28,31,33] appeared to have a greater impact on interoceptive sensibility than movement-based MBPs [24,36,41]. However, we note that Lauche et al. [24] was also the only RCT in this group, and only reported a mean increase in MAIA after tai chi on an exploratory basis, and as such did not conduct significance tests. The remaining trials were either secondary analyses, pilot, or longitudinal trials. We do hypothesize that mindfulness-based cognitive reappraisal skills may be an important component of any MBP for chronic pain, and that future yoga-based interventions for chronic pain would benefit from the explicit inclusion of such techniques such as acceptance and non-judgmental awareness of sensations, that may confer benefits beyond simply noticing sensations or proprioceptive awareness of physical alignment. Indeed, these techniques fall within the *yamas* and *niyamas,* the first and second limbs of Patanjali’s eight limb path of yoga, and include observances such as *aparigraha* (non-attachment) and *ahimsa* (non-violence or kindness) that may be an important component of interoceptive self-regulation.

Future studies examining yoga-based interventions for chronic pain would benefit from exploring manipulations to the intervention that would further explore the role of interoception. For example: instructor cuing can be manipulated to only include proprioceptive alignment cueing (i.e. align the knee over the heel) vs. also adding interoceptive cuing (i.e. noticing changes in the breath while in a posture or where sensation arises); participants can be instructed to link movements to their breath vs. noticing and practicing acceptance of sensations during sustained physical postures; or the intervention environment can be manipulated such that practicing without mirrors may challenge individuals to direct attention towards internal sensations rather than noticing how they appear in the mirror. It would also be beneficial to compare interoceptive outcomes in response to interventions that compare yoga to a seated MBP and/or other movement-based practices (e.g. stretching). Additionally, the MBP literature would benefit from evaluating both interoceptive sensibility and interoceptive accuracy measures, such as heartbeat tracking tasks, as changes in interoceptive sensitivity to internal sensations may be distinct from the subjective interpretation of those sensations [66].

## Conclusion

The literature linking yoga, interoception and pain remains limited and warrants further research. Findings from the broader mind-body literature indicate interoceptive techniques including attending to and acceptance of bodily sensations, including pain, may be key therapeutic mechanisms for chronic pain. Within this review, we identified no studies that linked interoceptive accuracy, pain and MBP, a topic that remains to be explored. Future yoga-based interventions would benefit examining both self-reported and interoceptive accuracy outcomes and from exploring the role of interoceptive techniques practiced during *asana*, in addition to structured *pranayama* and *dhyana* practices, to evaluate the role of interoception as a pain-modulating benefit of yoga.

## Supplementary Material

Supplemental Material

## Data Availability

The detailed study protocol is available upon request.
