# Supplementary material for: Mind-body practices, interoception and pain: a scoping review of behavioral and neural correlates
Source: Ann Med. 2023 Nov 8;55(2):2275661. doi: 10.1080/07853890.2023.2275661 (PMC10768869; doi:10.1080/07853890.2023.2275661)
Supplement: Supplemental Material [file IANN_A_2275661_SM2317.docx]

**Supplementary Material**

**Original Search String – June 2022**

The original search string included one comprehensive string, constructed from three clusters of terms representing the interoception, mind-body practice, and pain bodies of literature.

(“interoception” OR “interoceptive awareness” OR “interoceptive accuracy” OR “interoceptive sensibility” OR “interoceptive sensitivity” OR “heartbeat tracking” OR “heartbeat detection” OR “body responsiveness” OR “body awareness” OR “body consciousness” OR “body connection” OR “emotional awareness” OR “emotional regulation” OR “affect regulation” OR “emotional experience” OR “attention regulation” OR “embodied” OR “embodiment” OR “mind-body connection” OR “somatosensory cortex” OR “somatomotor cortex” OR “insula” OR “insular cortex” OR “anterior cingulate cortex” OR “prefrontal cortex” OR “ventromedial prefrontal cortex” OR “dorsolateral prefrontal cortex”) AND (“yoga” OR “yogic” OR “pranayama” OR “breathing” OR “mindfulness” OR “meditation” OR “contemplative” OR “mind-body”) AND (“pain” OR “chronic pain” OR “fibromyalgia” OR “migraine”)

**Updated Search String to include Tai chi and Qigong – July 2023**

The updated search string included the original terms above, with the addition of tai chi and qigong in the mind-body practice cluster of terms.

(“interoception” OR “interoceptive awareness” OR “interoceptive accuracy” OR “interoceptive sensibility” OR “interoceptive sensitivity” OR “heartbeat tracking” OR “heartbeat detection” OR “body responsiveness” OR “body awareness” OR “body consciousness” OR “body connection” OR “emotional awareness” OR “emotional regulation” OR “affect regulation” OR “emotional experience” OR “attention regulation” OR “embodied” OR “embodiment” OR “mind-body connection” OR “somatosensory cortex” OR “somatomotor cortex” OR “insula” OR “insular cortex” OR “anterior cingulate cortex” OR “prefrontal cortex” OR “ventromedial prefrontal cortex” OR “dorsolateral prefrontal cortex”) AND (“yoga” OR “yogic” OR “pranayama” OR “breathing” OR “mindfulness” OR “meditation” OR “contemplative” OR “mind-body” OR “tai chi” OR “qigong”) AND (“pain” OR “chronic pain” OR “fibromyalgia” OR “migraine”)
